# Stronger Together: Isograft Fusion Accelerates Recovery and May Facilitate Heat‐Evolved Symbiont Transmission in Bleached Adult Corals

**DOI:** 10.1111/1758-2229.70418

**Published:** 2026-10-02

**Authors:** Bede G. Johnston, Catalina Parra V., Matthew R. Nitschke, Wing Yan Chan, Madeleine J. H. van Oppen

**Affiliations:** ^1^ Australian Institute of Marine Science Townsville Queensland Australia; ^2^ School of BioSciences The University of Melbourne Parkville Victoria Australia; ^3^ School of Biological Sciences, Victoria University of Wellington Wellington New Zealand; ^4^ Department of Biochemistry and Pharmacology The University of Melbourne Parkville Victoria Australia

**Keywords:** coelenteron, coral bleaching, coral fusion, coral restoration, experimental evolution, free‐living symbionts, gastrovascular canals, heat‐evolved, horizontal transmission, photophysiology

## Abstract

Experimentally evolved, heat‐tolerant algal symbionts (heat‐evolved; HE) offer a promising means of enhancing coral holobiont thermotolerance under warming oceans. However, translating their benefits into restoration practices requires scalable delivery methods. Coral tissue fusion may provide one such pathway by facilitating HE symbiont transfer to wild corals; however, its feasibility remains largely untested. As an initial test, we paired adult isografts of *Galaxea fascicularis* and *Psammocora columna* hosting HE *Cladocopium proliferum* (SS8) with chemically bleached, SS8‐naive recipients. Fusion was first observed after 3 days in *G. fascicularis* and 9 days in *P. columna*. In both species, fusion was followed by increased pigmentation and photochemical efficiency at the recipient's fusion interface relative to distal tissue and unfused controls. After ~50 days, SS8 was detected at low levels (< 3.5%) in 15/19 fused *G. fascicularis* recipients, although detection was also common among unfused horizontal‐transmission controls maintained in the same water column (13/18). These findings provide the first empirical evidence that conspecific coral tissue fusion is associated with rapid localised physiological recovery and can coincide with HE symbiont acquisition, but also highlight the need to distinguish tissue‐mediated transfer from background horizontal transmission. Fusion may therefore represent a complementary pathway for symbiont delivery in assisted‐evolution frameworks.

## Graphical abstract

This study investigates whether tissue fusion between adult corals enables transmission of heat‐evolved (HE) symbionts. After ~50 days of fusion to a donor, HE symbionts were detected in chemically bleached *Galaxea fascicularis* recipients. Fusion also accelerated post‐bleaching recovery. These results have promising implications for large‐scale dissemination of HE symbionts.

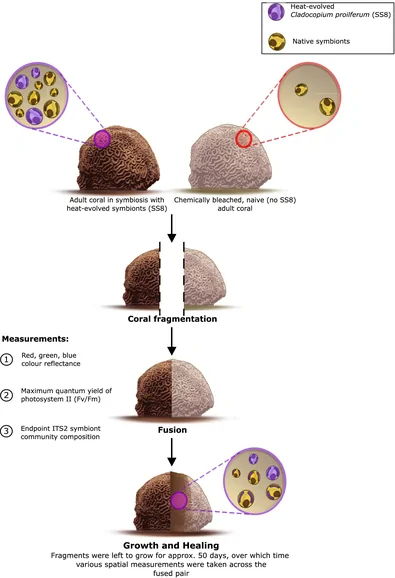

## 1 Introduction

The success of coral reef ecosystems is underpinned by the symbiotic relationship between reef‐building corals and endosymbiotic dinoflagellates (family Symbiodiniaceae) (Muller‐Parker et al. 2015). Residing within coral gastrodermal cells, these photosynthetic, microalgal symbionts provide the coral host with most of its nutritional requirements by converting inorganic carbon and host‐derived waste products into energy‐rich photosynthates (Muscatine and Weis 1992; Davy et al. 2012; Lipschultz and Cook 2002; Wiedenmann et al. 2023; Muscatine and Porter 1977). Although highly efficient, this metabolic exchange can become unstable under stressful environmental conditions, such as elevated temperatures or excessive light exposure, leading to host energetic stress, and, if stress persists, degradation or expulsion of the algal symbionts—that is, coral bleaching (Weis 2008; Hoegh‐Guldberg 1999).

The ability of corals to tolerate and recover from environmental stress is strongly influenced by the physiological performance of their resident Symbiodiniaceae (Weis 2008; Cunning and Baker 2020; Turnham et al. 2023). For example, corals associated with naturally thermotolerant symbionts, including some *Durusdinium* spp., often exhibit improved bleaching tolerance under elevated temperatures (Turnham et al. 2023; Palacio‐Castro et al. 2023; LaJeunesse et al. 2014; Hoadley et al. 2019; Berkelmans and van Oppen 2006; Silverstein et al. 2015). However, such associations have also been linked to context‐dependent physiological trade‐offs (Little et al. 2004; Cantin et al. 2009; Cunning et al. 2015; Matsuda et al. 2023), but see Turnham et al. (2023), Kemp et al. (2023) Yuyama and Higuchi (2014) and may still fail to prevent bleaching during severe marine heatwaves (Claar et al. 2020). These limitations have motivated the development of symbiont manipulation approaches that enhance coral thermal tolerance while minimising trait trade‐offs.

One promising approach is the experimental evolution of Symbiodiniaceae under thermal selection, whereby cultured symbiont populations are subjected to prolonged elevated temperatures to generate strains with improved heat tolerance (van Oppen et al. 2015; Nitschke et al. 2024). Using a thermal ratchet design (Huertas et al. 2011), Chakravarti et al. (2017), Chakravarti and van Oppen (2018) generated several heat‐evolved (HE) strains of *Cladocopium proliferum* that exhibited enhanced growth and photochemical efficiency under acute heat stress relative to their wild‐type (WT) counterparts. For some HE strains, these benefits have been shown to extend to the coral holobiont, with no observable trait trade‐offs (Chan et al. 2023; Quigley et al. 2023; Buerger et al. 2020).

The integration of HE symbionts into reef restoration practices requires scalable delivery methods that promote reliable uptake and persistence within coral hosts (Nitschke et al. 2024; Johnston et al. 2025). Adult coral tissue fusion may offer one such pathway by creating direct tissue continuity between colonies through which symbionts could potentially move across the fusion boundary. However, whether fusion can facilitate symbiont movement between adult corals remains largely untested, with no previous demonstration involving experimentally evolved heat‐tolerant strains.

Coral tissue fusion is a well‐documented and ecologically important life‐history strategy observed across multiple life stages. It occurs frequently among gregariously settled larvae (Amar et al. 2008; Hidaka 1985; Jiang et al. 2015; Ligson et al. 2022), closely related juveniles (Raymundo and Maypa 2004; Forsman et al. 2006; Hidaka et al. 1997), and genetically identical adult fragments (Rinkevich and Loya 1983; Page et al. 2018; Chornesky 1991), with some evidence also reported for allogeneic fusion between genetically distinct conspecifics (Frank and Rinkevich 1994; Nozawa and Loya 2005). Fusion has been shown to confer a number of physiological benefits, including accelerated growth, expanded spatial coverage, and earlier onset of sexual maturity (Raymundo and Maypa 2004; Rinkevich and Loya 1983; Page et al. 2018). These benefits are thought to arise, at least in part, through integration of the colonies' gastrovascular systems (GVS), which support gas exchange, extracellular digestion, waste removal, and the distribution of dissolved and particulate organic matter throughout the colony (Gateno et al. 1998).

Beyond organic matter and nutrients, intact Symbiodiniaceae cells have been observed circulating within the coral coelenteron (gastrovascular cavity; GVC) (Gateno et al. 1998; Gladfelter 1983). Although such cells are generally thought to be digested or expelled via the polyp mouth (Davy et al. 2012; Titlyanov et al. 1996; Stimson and Kinzie III 1991), gastrovascular integration following fusion could instead allow donor‐derived symbionts to enter the recipient GVC, where they may be captured and internalised by ciliated endodermal cells, as observed in Hirose et al. (2008). If retained, these cells could establish within recipient tissues, thereby providing a biologically plausible pathway through which fusion may promote symbiont uptake.

This study investigates whether adult isograft tissue fusion between a donor fragment hosting HE *C. proliferum* (strain SS8) and a chemically bleached recipient fragment can facilitate detectable acquisition of SS8. To test this, donor and recipient fragments were generated from two coral species, *Galaxea fascicularis* and *Psammocora columna*. Fragments were cut to expose adjacent tissue margins, positioned in direct contact with paired conspecific fragments, and maintained for ~50 days, during which fusion progression, tissue repigmentation, and photochemical efficiency were regularly assessed. At the conclusion of the experiment, tissue samples were collected from defined positions relative to the fusion boundary and analysed using Internal Transcribed Spacer 2 (ITS2) amplicon sequencing to detect SS8 where ITS2 profiles permitted strain‐level resolution. Together, this approach provides a controlled first test of whether coral tissue fusion can facilitate symbiont acquisition between adult coral fragments.

## 2 Materials and Methods

### 2.1 Collection and Maintenance of *Galaxea fascicularis* and Psammocora Columna Colonies

Three colonies of *G. fascicularis* (herein referred to as gA, gB, and gC) were collected from Davies Reef in the central Great Barrier Reef (GBR) on October 29, 2021, at depths of 5–10 m (collection permit: G12/35236.1). In addition, four colonies of *P. columna* (pA, pB, pC, and pD) were collected from Fantome Reef in the central GBR on May 25, 2022, at a depth of ~3 m (collection permit: G21/38062.1). Distances between individual colonies were > 5 m and are thus assumed to represent distinct genotypes. All colonies were transported to the National Sea Simulator at the Australian Institute of Marine Science, Townsville, and acclimated for 2 weeks. Following acclimation, *G. fascicularis* colonies were sectioned into 4–5 polyp fragments using a diamond blade band saw (Gryphon C‐40 Bandsaw) and affixed to 22 × 30 mm PVC tiles using Gorilla Super Glue. *P. columna* colonies were cut into ~3 cm^2^ fragments and affixed to aragonite frag plugs (Large OW100LCFP, Aquasonic, Wauchope, Australia). Fragments were maintained in 2‐μm filtered seawater (FSW) with a continuous FSW turnover rate of 5 L h^−1^. Corals were maintained under an 11‐h photoperiod (06:50–17:50) provided by Hydra 64HD LED lights (Aqua Illumination, Bethlehem, PA, USA), delivering within‐tank light intensities ranging from 102 to 180 μmol photons m^−2^ s^−1^. Corals were fed daily with *Artemia* nauplii (0.5 nauplii mL^−1^). Filamentous algae and biofilm were manually removed from tiles and plugs 1–2 times per week. Water temperature was maintained at 26.7°C–27.0°C. Water movement and aeration were provided using air stones.

### 2.2 Heat‐Evolved Symbiont (SS8)

The heat‐evolved symbiont used was *Cladocopium proliferum* (SCF 055.01.08; referred to as SS8 as per (Chan et al. 2023; Quigley et al. 2023; Buerger et al. 2020; Johnston et al. 2025)). Cultures of SS8 were maintained at 31°C under a light intensity of 40 to 70 μmol photons m^−2^ s^−1^ with a 12:12 h light–dark cycle. The cultivation medium used was 1% standard IMK culture medium (Nihon Pharmaceutical Co.). Cultures were refreshed every 4 weeks, involving the replacement of more than 50% of the existing culture medium with fresh IMK medium.

### 2.3 Generation of Coral Donors and Recipients

SS8‐ *G. fascicularis* and SS8‐*P. columna* donors were generated via chemical bleaching followed by inoculation with cultured SS8. The chemical bleaching protocol was adapted from Scharfenstein et al. (2022) and refined through preliminary trials to balance sufficient symbiont reduction with host tissue integrity and survivorship. The selected menthol‐diuron exposure regime induced visible paling and reduced photochemical signal while avoiding extensive tissue loss or mortality. Briefly, a subset of coral fragments from both species was incubated in 5 L of FSW supplemented with menthol (0.39 mM) and diuron (0.13 μM) for 8 h daily (08:30–16:30), during which gentle aeration was provided using air stones. Following incubation, menthol‐diuron‐spiked FSW was removed, and fragments were returned to FSW with regular turnover (5 L h^−1^) for 16 h (16:30–08:30). This cycle was repeated for four consecutive days, followed by a three‐day recovery period, together constituting one round of chemical bleaching. For *P. columna*, one round was applied, comprising four treatment days followed by three recovery days. For *G. fascicularis* , two complete rounds were applied consecutively, comprising eight treatment days and two three‐day recovery periods, for a total bleaching schedule of 14 days. The number of rounds was determined from preliminary trials and visual assessment under a high‐power stereomicroscope, with *P. columna* sufficiently bleached after one round and *G. fascicularis* requiring a second. No feeding occurred during the treatment or recovery periods.

Chemically bleached *G. fascicularis* and *P. columna* fragments were then inoculated daily with cultured SS8 (final cell density: 10,000 cells mL^−1^) in line with previous coral–Symbiodiniaceae inoculation approaches (Scharfenstein et al. 2022). Inoculations were performed during the culture's peak motile period (~1 h after the culture incubator lights turned on) over a seven‐day period. Corals were then allowed to repigment for 7 weeks. Recovery was monitored via image‐based red, green, and blue (RGB) reflectance analysis and dark‐adapted maximum quantum yield of photosystem II (*F*_*v*_/*F*_*m*_) (details provided below). ITS2 community composition analysis confirmed the presence of SS8 in donor fragments (details provided below). To generate coral recipients for subsequent tissue fusion, the remaining *G. fascicularis* and *P. columna* fragments underwent the same chemical bleaching procedure, timed such that they were sufficiently bleached by the end of the donors' seven‐week recovery period.

### 2.4 Coral Tissue Fusion

#### 2.4.1 Experimental Treatments

To generate donor and recipient isografts, corals were first detached from their PVC tiles or aragonite plugs using a diamond‐blade band saw (Gryphon C‐40). Recipient fragments were cut perpendicularly to the base. Each fragment was first halved, and one half was then divided again to produce three pieces: one larger half‐fragment and two smaller quarter‐fragments (Figure 1A). The larger piece was assigned to the fused recipient (FR) treatment. The two smaller pieces remained unfused and were allocated to control treatments: a horizontal transmission control (HT) placed in the same tank as SS8 donor fragments and fused donor–recipient pairs; and a symbiont‐exclusion control (EC) maintained in a separate tank without exposure to external SS8 symbionts. Donor fragments were similarly bisected to produce two half‐fragments: one assigned to the fused donor (FD) treatment and the other to the unfused donor (UD) control, which was maintained in the same tank but kept physically separate from all recipient fragments.

**FIGURE 1:**
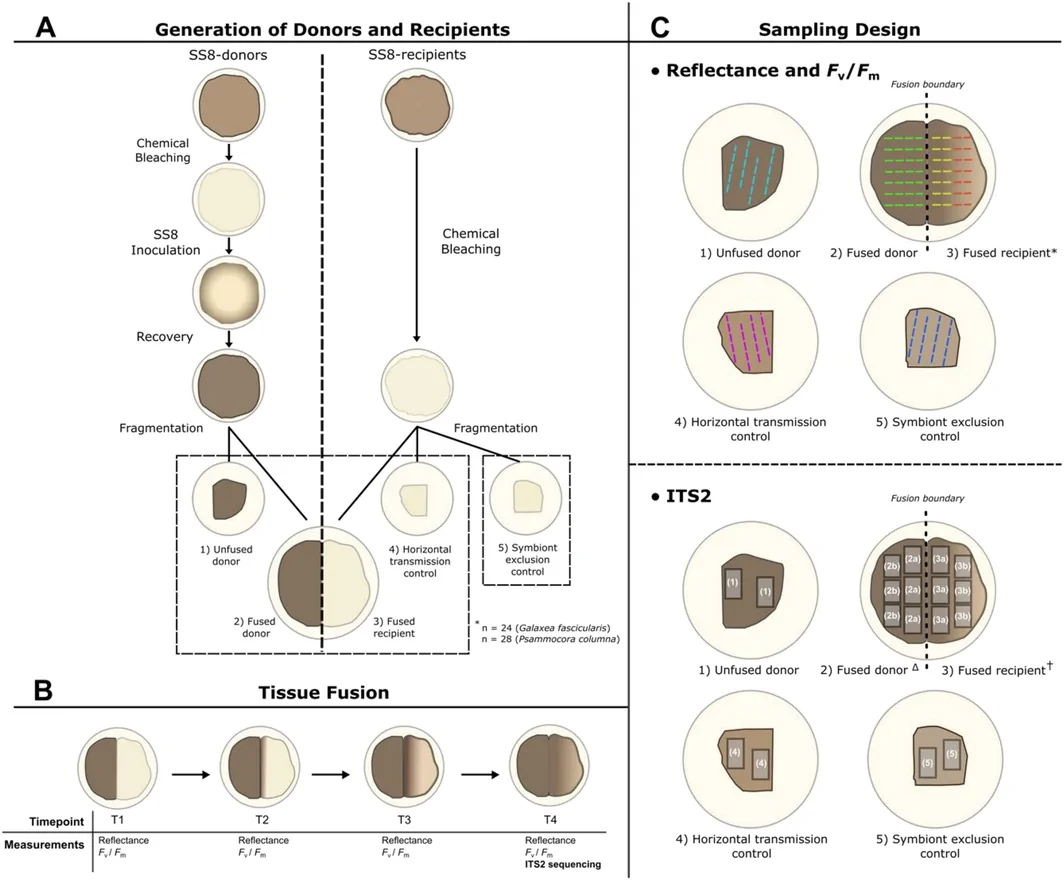
Schematic overview of the experimental design used to assess coral‐to‐coral transmission of the heat‐evolved *Cladocopium proliferum* strain SS8 in *Psammocora columna* and *Galaxea fascicularis* . (A) SS8‐donor corals were generated via chemical bleaching and inoculation with SS8, followed by a recovery period. SS8‐recipient corals were generated by chemically bleaching fragments from the same source colonies immediately prior to the experiment. Donor and recipient fragments were cut using a band saw and allocated to one of five treatments: (1) unfused donor (UD), maintained independently; (2) fused donor (FD), the donor side of fused donor‐recipient pairs; (3) fused recipient (FR), the recipient side of fused donor‐recipient pairs; (4) horizontal transmission control (HT), recipient fragments maintained in shared tanks alongside treatments 1–3; and (5) symbiont‐exclusion control (EC), recipient fragments maintained in isolated tanks devoid of external SS8 sources. Tank allocation is indicated by hatched boxes. (B) Coral photophysiology, measured as maximum quantum yield of photosystem II (*F*_*v*_/*F*_*m*_), and reflectance were measured at multiple timepoints throughout the experiment to examine spatial and temporal patterns of recipient recovery. Endpoint tissue samples were collected after ~50 days for Internal Transcribed Spacer 2 (ITS2) amplicon sequencing. (C) Sampling design for reflectance, *F*_*v*_/*F*_*m*_, and ITS2 analysis. *Top*: For UD, HT, and EC fragments, a minimum of four transects were drawn across the tissue surface in ImageJ (Schindelin et al. 2012). For fused donor–recipient pairs, at least seven transects were positioned perpendicular to the fusion line, with each midpoint centred on the line. *For the fused recipient, transects were subdivided into two spatial zones: Recipient‐boundary (yellow) and recipient‐far (red), allowing for the detection of localised variation in recovery. *Bottom*: Endpoint tissue sampling for ITS2 sequencing followed the same spatial structure. For UD, HT, and EC fragments, two technical replicates were collected from each fragment, one from the top surface and one from the side. For fused treatments 2^∆^ (FD) and 3^†^ (FR), samples were collected from defined spatial zones: (a) tissue adjacent to the fusion line (termed donor boundary and recipient boundary), and (b) tissue located distally from the line (donor far and recipient far), to capture spatial variation in symbiont identity across the fusion interface. Technical replicates sharing the same number or number–letter identifier were extracted separately, and the resulting DNA extracts were pooled before PCR amplification and library preparation.

Immediately following sectioning, donor and recipient fragments were placed in their respective tanks and allowed to recover for 4 days before fusion setup. This recovery period was included to reduce the likelihood that symbionts released during cutting and acute tissue damage would contribute to waterborne acquisition during the initial fusion period. After this recovery period, *G. fascicularis* and *P. columna* fragments from the same genotype—assigned to the FR and FD treatments—were carefully aligned on a new PVC tile or aragonite plug. To maximise tissue contact, the cut lateral margins—comprising both tissue and underlying skeleton—were positioned flush against one another and secured using super glue to promote tissue fusion (see Figure S1).

#### 2.4.2 Tank Allocation

A total of four 50‐L tanks were used for the experiment, with two tanks allocated to each species. For each species, one tank housed all SS8‐exposed treatments, including FR, FD, HT, and UD fragments, while the second housed EC fragments that were not exposed to external SS8 symbionts. Thus, the *G. fascicularis* and *P. columna* experiments each comprised one SS8‐exposed tank and one EC tank.

Each donor–recipient pairing and its corresponding unfused control fragments were derived from the same source genotype and treated as a biological replicate. For *G. fascicularis* , each treatment included eight replicate fragment sets per genotype across three genotypes (*n* = 24 per treatment), while for *P. columna*, each treatment included seven replicate fragment sets per genotype across four genotypes (*n* = 28 per treatment). Corals were maintained in their respective tanks for ~50 days, with physiological measurements taken at regular timepoints throughout the experiment and ITS2 samples collected at its conclusion (Table 1). Tank conditions and coral husbandry followed the procedures described above.

**TABLE 1:** Sampling schedule for physiological and molecular measurements in *Galaxea fascicularis* and *Psammocora columna*.

Species	Measurement	T1	T2	T3	T4
*Galaxea fascicularis*	Reflectance	5	15	29	50
	*F*_*v*_/*F*_*m*_	4	15	28	50
	ITS2	—	—	—	51
*Psammocora columna*
	Reflectance	5	16	33	48
	*F*_*v*_/*F*_*m*_	7	16	33	46
ITS2	—	—	—	50

*Note:* Timepoints (T1–T4) are reported as days post‐fusion setup (DPF) with 0 DPF defined as the day donor and recipient fragments were placed in direct contact to initiate fusion.

### 2.5 Coral Reflectance Analysis

Coral RGB reflectance values, used here as a proxy for bleaching severity, were quantified in ImageJ (Schindelin et al. 2012) using a transect‐based approach. Photographs of each coral fragment were taken at designated timepoints (Table 1) using a Nikon D810 camera in aperture‐priority mode (f/8, ISO 640, 1/80 s), with flash illumination provided by a Godox V860III Speedlite. Standardised lighting and positioning were maintained across timepoints to minimise variation. To calculate RGB reflectance for UD, HT, and EC treatments, a minimum of four transects were placed across each fragment (Figure 1C; dashed lines). Each transect spanned ~75% of the fragment's width to capture intra‐fragment variation while excluding areas affected by uneven lighting due to fragment geometry. RGB values were extracted from every pixel along each transect and averaged to yield mean values per transect for each RGB channel. One pixel corresponded to 0.035 mm.

For FR and FD, a minimum of seven transects were placed per fused pair, each spanning 60%–75% of the total fused fragment width and oriented perpendicular to the fusion line (Figure 1C). Each transect was centred over the visible fusion interface to enable targeted analysis of donor and recipient tissue along a consistent spatial axis. To minimise potential misclassification at the immediate fusion line where tissue origin may be ambiguous, the central 5 pixels on either side of the midpoint were excluded from analysis (~0.35 mm total). The remaining pixels were categorised into three spatial zones: (a) recipient boundary (6–55 pixels left of centre), (b) recipient far (56–105 pixels left of centre), and (c) donor (6–105 pixels right of centre). Mean reflectance values were calculated separately for each zone to capture localised variation across the fusion interface. RGB values were calibrated in ImageJ using six reference standards with known reflectance values. For each RGB channel, a fourth‐degree polynomial regression was applied to generate calibration curves. To account for minor lighting variation across timepoints, separate calibration curves were generated for each sampling interval. All reflectance values reported in this study are based on these calibrated measurements.

### 2.6 Maximum Quantum Yield of PSII (*F_v_*/*F*_*m*_)

The photochemical efficiency of coral fragments across all treatments was assessed using an imaging pulse‐amplitude modulation fluorometer (IMAGING‐PAM, Maxi version, Walz) (see Figure S2 for representative image). Corals were dark‐adapted for 15 min under low‐light conditions (< 5 μmol photons m^−2^ s^−1^) prior to measurement. Maximum quantum yield of photosystem II (*F*_*v*_/*F*_*m*_ = [*F*_*m*_ − *F*_*0*_]/*F*_*m*_) was measured between 11:00 and 12:00 at designated timepoints (Table 1). The same transect‐based approach described for reflectance analysis was applied to extract *F*_*v*_/*F*_*m*_ values across each coral fragment (Figure 1C). Imaging‐PAM parameters were set as follows: measuring light intensity = 4, gain = 3, frequency = 4, and damping = 2. Data were collected using ImagingWin software (V2.32 FW Multi RGB; Walz GmbH, Effeltrich, Germany).

Physiological measurements were conducted at four approximately matched timepoints, with slight differences between species and measurement types, followed by endpoint ITS2 sampling (Table 1). For *G. fascicularis* , reflectance was measured at 5, 15, 29, and 50 DPF, *F*_*v*_/*F*_*m*_ at 4, 15, 28, and 50 DPF, and ITS2 samples were collected at 51 DPF. For *P. columna*, reflectance was measured at 5, 16, 33, and 48 DPF, *F*_*v*_/*F*_*m*_ at 7, 16, 33, and 46 DPF, and ITS2 samples were collected at 50 DPF.

### 2.7 Macro‐Photography

A series of high‐resolution close‐up images (0.63× to 4× magnification) of all fusion pairings were captured using a Leica MZ10F stereomicroscope equipped with a DFC450 C digital camera. Images were collected as a detailed time series documenting fusion progression, including the timepoint at which fusion was first confirmed. Fusion was deemed successful when the tissues of adjoining fragments had visibly merged at the interface and their tissue origins could no longer be distinguished. Illumination for imaging was provided by an EL6000 external light source and a dual‐arm LED gooseneck.

### 2.8 Sampling Design

For each fragment pairing (encompassing treatments 1–5), a total of seven sample types were collected: (1) UD, (2a) FD boundary, (2b) FD far, (3a) FR boundary, (3b) FR far, (4) HT, and (5) EC (see Figure 1C). To generate sample types 2a/b and 3a/b, each fused coral pair was first sectioned along the visible fusion line to separate the donor and recipient fragments (see Figure S3). To minimise cross‐contamination between donor and recipient tissues, ~1 mm of tissue and underlying skeleton was shaved from the adjoining fusion interface of both fragments and discarded. A second longitudinal cut was then made ~0.5 mm from the shaved edge to isolate the donor and recipient boundary samples. Boundary samples therefore represented tissue immediately adjacent to, but not directly on, the fusion interface. Following removal of the boundary samples, an additional longitudinal cut was made ~5 mm from the newly exposed margin to obtain the donor and recipient far samples, representing tissue distal to the fusion boundary.

To minimise cross‐contamination, cutting tools were cleaned between samples by sequential dipping in ethanol, rinsing in freshwater and 0.2 μm FSW, and drying before reuse. Tissue sections from sample types 2a/b and 3a/b were trimmed into three ~match‐head‐sized pieces. For sample types 1, 4, and 5, two ~match‐head‐sized pieces were taken from the top and side surfaces of each coral fragment. All tissue pieces were rinsed in 0.2 μm FSW and preserved in absolute ethanol for subsequent DNA extraction. For sample types 2a/b and 3a/b, DNA extracted separately from the three tissue pieces was pooled prior to PCR amplification and ITS2 sequencing.

### 2.9 DNA Extraction and ITS2 Sequencing

Details on DNA extraction, PCR amplification and library preparation are provided in Supporting Information. Paired‐end sequencing (2 x 300 bp) was performed on an Illumina NextSeq 2000 at the Walter and Eliza Hall Institute, Melbourne. Demultiplexed raw sequences were submitted to SymPortal for ITS2 profiling (Supporting Information). SymPortal identifies recurring combinations of ITS2 sequence variants across samples as ITS2 type profiles, which represent putative Symbiodiniaceae taxa. Individual sequences that define these profiles are referred to as defining intragenomic variants (DIVs). Sequencing data were downloaded from SymPortal and analysed in R version 4.2.0 (R Core Team 2022).

The ITS2 type profile of the SS8 culture is C1‐C1b‐C42.2‐C1bh‐C1c‐C1br‐C1cb‐C1ge‐C1w‐C3ju‐C72k‐C1al‐C42ca‐C3sa‐C42ao‐C1jx‐C1cu (Scharfenstein et al. 2022). Due to the relatively low abundance of SS8 in experimental corals, putative SS8 presence was primarily inferred from the dominant SS8‐associated DIVs (e.g., C1‐C1b‐C42.2). These DIVs provided sufficient resolution to assess putative SS8 acquisition in *G. fascicularis* . In contrast, ITS2 data from *P. columna* were excluded from strain‐level symbiont analyses because high sequence similarity between SS8 and homologous *Cladocopium* spp. associated with this host precluded reliable discrimination of SS8. Accordingly, ITS2‐based evidence of putative SS8 acquisition was restricted to *G. fascicularis* .

### 2.10 Statistical Analysis

All statistical analyses were conducted in R (v4.3.1). For both *P. columna* and *G. fascicularis* , principal component analysis (PCA) was performed on the RGB reflectance values extracted from linear transects across coral tissue. PC1 accounted for > 92% of the total variance in both species and was therefore used as the response variable in downstream reflectance analyses. Changes in reflectance PC1 scores and *F*_*v*_/*F*_*m*_ over time and among experimental categories were assessed using linear models and linear mixed‐effects models. Paired *t‐*tests were used to compare pre‐ and post‐bleaching samples. For post‐bleaching timepoints, model structure was selected using Akaike Information Criterion (AIC) comparisons. Genotype was included as a fixed effect and fusion pairing as a random effect. Pairwise comparisons were conducted using estimated marginal means with Tukey adjustment.

## 3 Results

### 3.1 Fusion Outcomes

Successful tissue fusion was achieved in all donor–recipient pairings: 24 out of 24 in *Galaxea fascicularis* and 28 out of 28 in *Psammocora columna*. Fusion was first observed as early as day three in *G. fascicularis* and day nine in *P. columna* (Figures 2A,B and 3A,B respectively; see Table S1 for the full list of fusion onset times). Although initial tissue integration occurred rapidly, complete integration along the tissue boundary progressed more gradually. This slower progression appeared to reflect minor misalignments between donor and recipient margins, requiring additional tissue growth to bridge remaining gaps.

**FIGURE 2:**
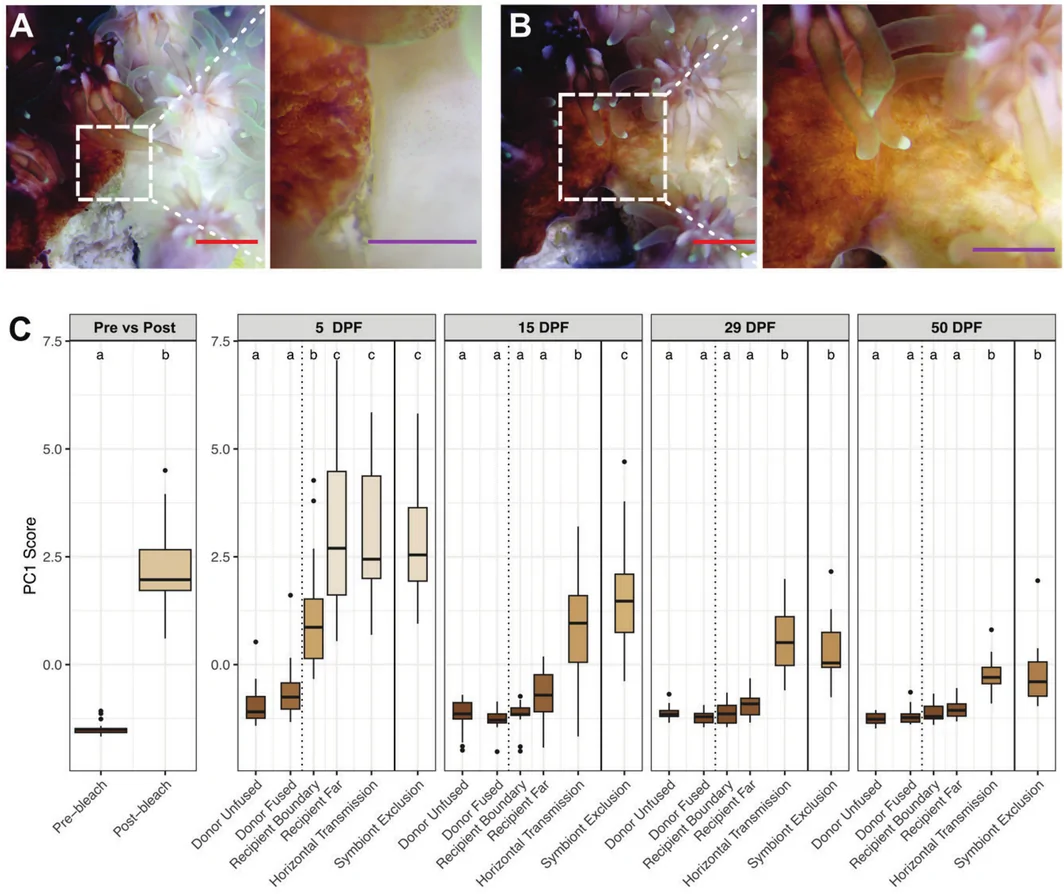
(A) *Galaxea fascicularis* donor (pigmented) and recipient (bleached) fragments at 0 DPF (days post‐fusion setup). (B) The same donor‐recipient pair at 6 DPF. Insets show magnified view of fusion line at both timepoints with clear tissue fusion observed at 6 DPF. Scale bars represent 2.5 mm (red) and 1 mm (purple). (C) Reflectance‐based principal component analysis (PCA) scores (PC1) for *G. fascicularis* across timepoints and experimental categories. Each box represents the distribution of PC1 scores within a category at a given timepoint. The leftmost plots (Pre vs. Post) compare coral reflectance profiles before and after chemical bleaching using a paired *t‐*test. Experimental timepoints post‐fusion track changes in PC1 scores over time across five experimental categories: Unfused donor, fused donor, recipient boundary, recipient far, horizontal transmission, and symbiont exclusion. The colour scale reflects the mean PC1 score for each category, bounded by the minimum and maximum values observed across all timepoints. Category differences at each timepoint were assessed using post hoc pairwise contrasts from the fitted model. Different letters denote statistically significant groupings (Tukey‐adjusted *p* < 0.05).

**FIGURE 3:**
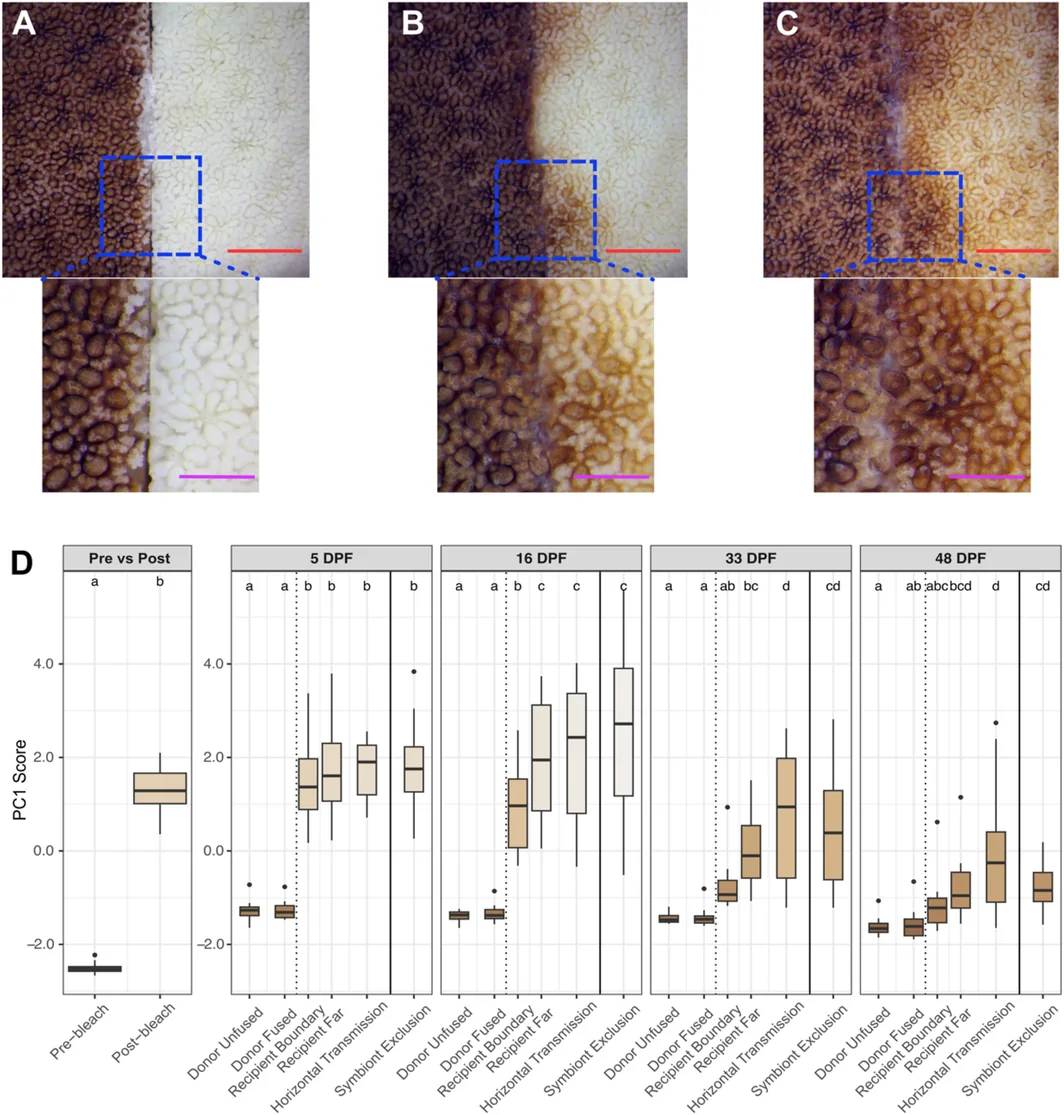
(A) *Psammocora columna* donor (pigmented) and recipient (bleached) fragments at 0 DPF (days post‐fusion setup). (B) The same donor–recipient pair at 13 DPF and (C) 36 DPF (these images do not align temporally with reflectance measurements). Insets in both panels show magnified views of the fusion line revealing clear tissue fusion by 13 DPF. Scale bars represent 5 mm (red) and 2.5 mm (purple). (D) Reflectance‐based principal component analysis (PCA) scores (PC1) for *P. columna* across timepoints and experimental categories. Each box represents the distribution of PC1 scores within a category at a given timepoint. The leftmost plots (Pre vs. Post) compare coral reflectance profiles before and after chemical bleaching using a paired *t*‐test. Experimental timepoints post‐fusion track changes in PC1 scores over time across the five experimental categories: Unfused donor (UD), fused donor (FD), recipient boundary, recipient far, and horizontal transmission (HT) and symbiont‐exclusion (EC) controls. The colour scale reflects the mean PC1 score for each category, bounded by the minimum and maximum values observed across all timepoints. Category differences at each timepoint were assessed using post hoc pairwise contrasts from the fitted model. Different letters denote statistically significant groupings (Tukey‐adjusted *p* < 0.05).

### 3.2 Reflectance‐Based Principal Component Analysis (PCA)

#### 3.2.1 *Galaxea fascicularis*

A strong bleaching‐associated shift in reflectance profiles was observed in *G. fascicularis* following chemical treatment, with mean PC1 scores increasing significantly from −1.49 ± 0.15 when fragments were fully pigmented (Pre‐bleach) to 2.20 ± 0.93 when tissue was visibly bleached (Post‐bleach) (*p* < 0.05, paired *t*‐test; Figure 2C). Lower PC1 scores therefore corresponded to greater pigmentation, while higher scores corresponded to bleached tissue. After bleaching was confirmed, recipient and donor fragments were assigned to their respective treatments, and reflectance was tracked over time. Linear mixed‐effects modelling revealed a significant treatment‐by‐timepoint interaction (*p* < 0.001), indicating divergent PC1 trajectories among experimental groups (Figure 2C).

Both FD and UD tissues maintained low, stable PC1 scores throughout the experiment, with no significant differences detected between treatments at any timepoint (*p* > 0.74). In contrast, all recipient treatments exhibited significantly elevated PC1 scores relative to donors at 5 days post‐fusion setup (DPF). At this timepoint, recipient boundary tissue showed moderately elevated scores (1.12 ± 1.33) that were significantly higher than both FD and UD (*p* < 0.0001), but lower than recipient far, HT, and EC (*p* < 0.01 for all). By 15 DPF, recipient boundary scores had declined sharply to −1.17 ± 0.33 and were statistically indistinguishable from both FD and UD (*p* > 0.99), a pattern that persisted through the end of the experiment. Recipient far tissue followed a similar trajectory, with PC1 scores declining from 3.04 ± 1.99 at 5 DPF to −0.75 ± 0.65 at 15 DPF, becoming significantly lower than HT and EC (*p* < 0.0001 for both) and statistically indistinguishable from FD, UD (*p* > 0.1), and recipient boundary tissue (*p* = 0.56). This convergence was maintained at 29 and 50 DPF.

By 15 DPF, PC1 scores had declined in both HT and EC treatments (HT: 0.72 ± 1.26; EC: 1.55 ± 1.34), with a significantly greater reduction observed in HT than EC (*p* = 0.0076). Scores declined further by 29 DPF (HT: 0.52 ± 0.84; EC: 0.30 ± 0.74), at which point the difference between treatments was no longer significant, although both remained elevated relative to all donor and recipient fusion zones. By 50 DPF, PC1 scores decreased further to −0.27 ± 0.42 (HT) and −0.28 ± 0.65 (EC), but remained significantly higher than donor and recipient fusion zones.

#### 3.2.2 Psammocora Columna

Following chemical bleaching, mean PC1 scores of *P. columna* increased significantly from −2.51 ± 0.11 (Pre‐bleach) to 1.31 ± 0.48 (Post‐bleach) (*p* < 0.05, paired *t*‐test; Figure 3D). Linear mixed‐effects modelling identified a significant treatment‐by‐timepoint interaction (*p* < 0.001), indicating divergent PC1 trajectories among experimental groups over time (Figure 3D).

Across all timepoints, FD and UD treatments exhibited the lowest PC1 scores, with no significant differences detected between the two donor treatments at any timepoint. In contrast, all recipient treatments displayed significantly elevated PC1 scores relative to donors at 5 DPF with no significant differences detected among recipient treatments (*p* > 0.05). At 16 DPF, recipient boundary PC1 scores had declined but remained significantly higher than donor treatments and lower than the other recipient treatments, placing recipient boundary tissue at an intermediate level. From 33 DPF onwards, recipient boundary PC1 scores declined further (−0.76 ± 0.52 at 33 DPF; −1.16 ± 0.55 at 48 DPF), and became statistically indistinguishable from both FD and UD treatments (*p* > 0.05). Recipient far tissue followed a similar less pronounced trajectory, with scores declining from 1.69 ± 0.94 at 5 DPF to −0.81 ± 0.68 at 48 DPF. At 16 DPF, recipient far tissue exhibited significantly higher PC1 scores than recipient boundary (2.00 ± 0.20 vs. 0.94 ± 0.20; *p* < 0.01), but remained lower than HT and EC treatments (*p* < 0.05).

By 33 DPF, recipient far tissue was statistically indistinguishable from both recipient boundary tissue (−0.07 ± 0.69 vs. −0.76 ± 0.52; *p* = 0.08) and EC treatments (0.39 ± 1.22; *p* = 0.16), but remained significantly lower than HT (0.76 ± 1.42; *p* < 0.05). At 48 DPF, recipient far PC1 scores (−0.81 ± 0.68) were statistically indistinguishable from FD (−1.58 ± 0.31; *p* = 0.12), HT (−0.14 ± 1.33; *p* = 0.16), and EC (−0.77 ± 0.49; *p* = 0.95), but remained significantly higher than UD (−1.63 ± 0.19; *p* = 0.03).

HT and EC corals exhibited the highest PC1 scores throughout the experiment, with no significant differences between treatments at any timepoint (*p* > 0.05). By 48 DPF, both remained significantly higher than UD and FD corals (*p* < 0.05), but were statistically indistinguishable from recipient far tissue. At this timepoint, EC was also statistically indistinguishable from recipient boundary tissue.

### 3.3 PSII Maximum Quantum Yield (*F_v_*/*F*_*m*_)

The maximum quantum yield of photosystem II (*F*_*v*_/*F*_*m*_) declined significantly in both *G. fascicularis* and *P. columna* fragments following chemical bleaching (*p* < 0.0001, paired *t*‐test). In *G. fascicularis* , *F*_*v*_/*F*_*m*_ declined by 30.5%, from 0.456 ± 0.036 to 0.317 ± 0.037, while in *P. columna*, it declined by 57.5%, from 0.478 ± 0.074 to 0.203 ± 0.055 (Figure 4A,B). Thus, the proportional decline in *F*_*v*_/*F*_*m*_ was almost twice as large in *P. columna* as in *G. fascicularis* . After bleaching, recipient and donor fragments were assigned to their respective treatments, and *F*_*v*_/*F*_*m*_ was tracked over time. Linear mixed‐effects modelling revealed a significant treatment‐by‐timepoint interaction for both species (*p* < 0.001), indicating distinct recovery trajectories across experimental groups (Figure 4A,B).

**FIGURE 4:**
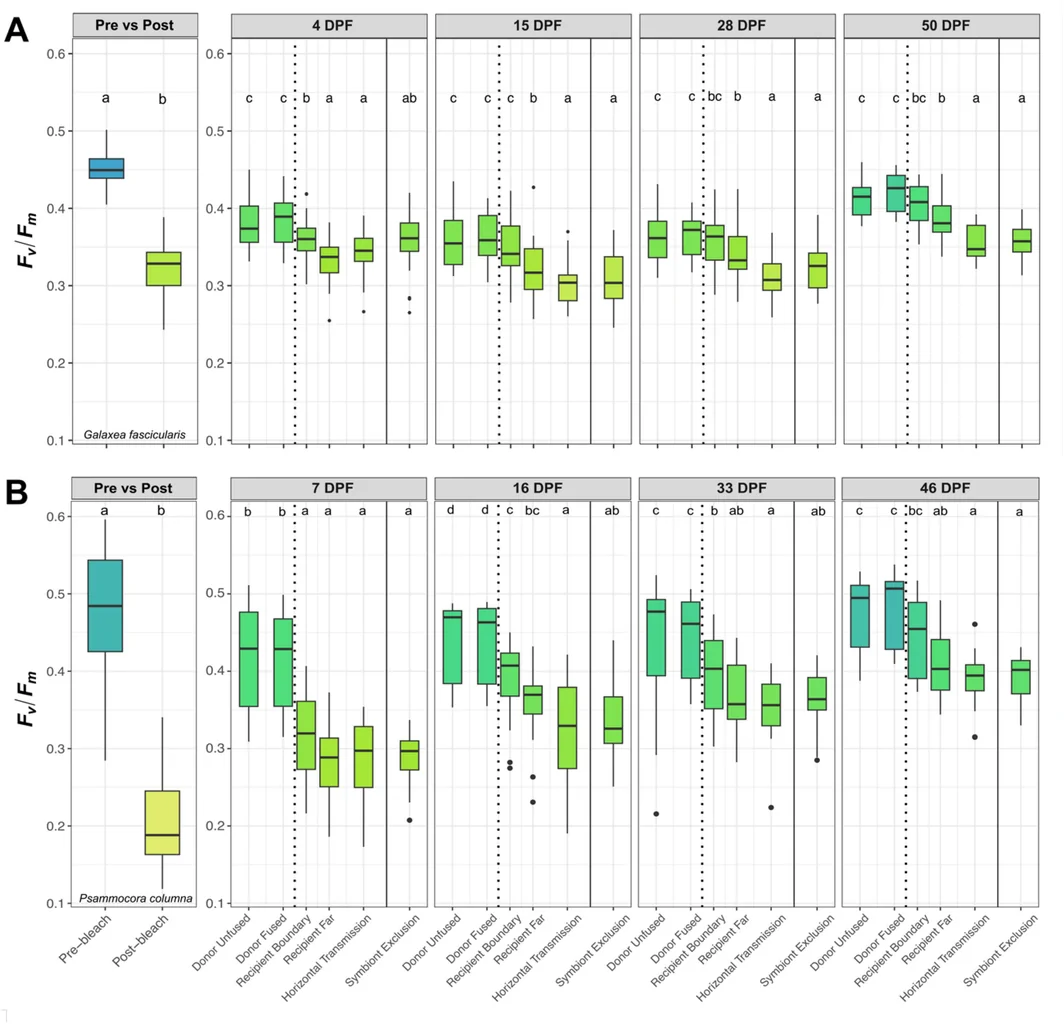
Maximum quantum yield of photosystem II (*F*_*v*_/*F*_*m*_) in (A) *Galaxea fascicularis* and (B) *Psammocora columna* across timepoints and experimental categories. Each boxplot displays the distribution of *F*_*v*_/*F*_*m*_ values for a given category at each timepoint. The leftmost plots (Pre vs. Post) compare *F*_*v*_/*F*_*m*_ values before and after chemical bleaching using a paired *t*‐test. All other timepoints represent post‐bleaching recovery, with differences between categories assessed using linear mixed‐effects models with fusion pairing specified as a random effect. The colour scale reflects the mean *F*_*v*_/*F*_*m*_ value for each category, bounded by the minimum and maximum values observed across all timepoints. Different letters indicate statistically distinct groups based on Tukey‐adjusted comparisons (*p* < 0.05).

In *G. fascicularis* , both FD and UD treatments had the highest *F*_*v*_/*F*_*m*_ values across all timepoints (0.362–0.422), with no significant differences detected between them. *F*_*v*_/*F*_*m*_ generally increased over time in both recipient boundary and recipient far tissues, although all treatments showed a slight decline between 4 and 15 DPF. Recipient boundary values increased from 0.362 ± 0.031 at 4 DPF to 0.404 ± 0.025 by the end of the experiment at 50 DPF (+11.6%), while recipient far increased from 0.333 ± 0.031 to 0.387 ± 0.026 (+16.2%). At 4 DPF, recipient boundary tissue exhibited significantly higher *F*_*v*_/*F*_*m*_ values than both recipient far and HT treatments but did not differ from EC. By 15 DPF, recipient boundary *F*_*v*_/*F*_*m*_ was significantly higher than all other recipient treatments and statistically indistinguishable from both donor treatments, a pattern that persisted across subsequent timepoints. Recipient far also recovered progressively, becoming statistically indistinguishable from recipient boundary tissue from 28 DPF onward, while remaining significantly lower than both donor treatments throughout the experiment.

HT and EC treatments consistently exhibited the lowest *F*_*v*_/*F*_*m*_ values throughout the experiment, except at 4 DPF when recipient far recorded the lowest value (0.333 ± 0.031). In HT, *F*_*v*_/*F*_*m*_ increased from a minimum of 0.302 ± 0.029 at 15 DPF to 0.355 ± 0.023 by 50 DPF (+17.5%). A similar increase was observed in EC corals, rising from 0.309 ± 0.037 at 15 DPF to 0.356 ± 0.023 at 50 DPF (+15.2%). No significant differences were detected between HT and EC treatments at any timepoint. Across all timepoints, both treatments exhibited significantly lower *F*_*v*_/*F*_*m*_ than recipient boundary tissue, except at 4 DPF when EC values did not differ significantly from recipient boundary.

In *P. columna*, FD and UD treatments consistently exhibited the highest *F*_*v*_/*F*_*m*_ values (0.414–0.477) across all timepoints, with no significant differences detected between them. Among recipient treatments, recipient boundary tissue recovered to the highest absolute *F*_*v*_/*F*_*m*_ value, increasing from 0.314 ± 0.054 at 7 DPF to 0.442 ± 0.052 at 46 DPF (+40.8%). At 16 and 46 DPF, recipient boundary *F*_*v*_/*F*_*m*_ was significantly higher than both HT and EC controls; at 33 DPF, it was significantly higher than HT but not EC. By 46 DPF, recipient boundary tissue was statistically indistinguishable from both donor treatments. Recipient far tissue followed a similar trajectory, increasing from 0.285 ± 0.046 to 0.406 ± 0.044 (+42.5%). Although consistently lower, recipient far tissue did not differ significantly from recipient boundary tissue at any timepoint and remained significantly lower than both donor treatments throughout the experiment.

HT and EC consistently exhibited the lowest *F*_*v*_/*F*_*m*_ values, except at 7 DPF when recipient far tissue recorded the lowest value (0.285 ± 0.046). Across the experiment, *F*_*v*_/*F*_*m*_ increased by 36% in HT (0.286 ± 0.053 to 0.389 ± 0.034), and by 35.2% in EC (0.290 ± 0.037 to 0.392 ± 0.031). No significant differences were observed between HT and EC treatments at any timepoint.

### 3.4 *Galaxea fascicularis* ITS2 Symbiodiniaceae Community Profiles

Prior to the start of the fusion experiment (0 DPF; ~50 days post‐SS8 inoculation), SS8 was detected in 3 of 19 *G. fascicularis* donor fragments, at low relative abundances of 0.68%, 0.84%, and 1.32%, with *Durusdinium* remaining dominant (> 98%; Figure S4). SS8 was not detected in any recipient fragments either before or after chemical bleaching (Figure S5). Bleaching substantially reduced the relative abundance of native *Cladocopium* in recipients. Of the five recipient fragments containing native *Cladocopium* prior to chemical bleaching, only three retained detectable levels afterwards, with mean relative abundance decreasing by 53.7% from 13.08% ± 4.68% to 6.06% ± 5.32% (mean ± SE).

Although SS8 was detected in only a subset of donor fragments sampled at 0 DPF, detection was more widespread in fused donor tissues by the final timepoint. SS8 was detected exclusively in donor far tissue (3/19), exclusively in donor boundary tissue (8/19), or in both regions (7/19), with relative abundances in FDs ranging from 0.94% to 5.65% (mean ± SE: 2.21% ± 0.21%) (Figure 5). No apparent differences in SS8 abundance were observed between donor boundary and donor far sampling regions (1.91% ± 0.23% vs. 2.41% ± 0.30%, respectively). Overall, SS8 was detected more frequently in FDs (18/19) than in UD corals (11/18; one UD coral was excluded due to insufficient sequencing depth). Patterns of SS8 co‐occurrence across donor, recipient, and HT categories are summarised in Table S2.

Among fused recipients whose paired donor had detectable SS8 at the final timepoint, SS8 was detected in 15 of 19 corals, occurring exclusively in recipient boundary tissue (2/15), exclusively in recipient far tissue (1/15), or in both (12/15). No apparent differences in SS8 abundance were observed between recipient boundary and recipient far sampling regions (1.59% ± 0.12% vs. 1.53% ± 0.16%, respectively). SS8 was also detected in 13 out of 18 HT fragments, where relative abundances ranged from 1.13% to 9.28% (mean ± SE = 3.54% ± 0.67%).

Across all treatments, only symbionts belonging to the genera *Cladocopium* and *Durusdinium* were detected, with *Durusdinium* remaining dominant. Residual native *Cladocopium* was detected in only a small subset of corals (4/19), spanning 11 distinct tissue sampling regions, with a mean relative abundance of 12.63% ± 6.43%—slightly higher than that observed in recipients immediately post‐bleaching. No SS8‐associated DIVs were detected in EC corals, suggesting no detectable cross‐contamination prior to or during the experiment.

Across all samples, SS8 relative abundance was significantly influenced by treatment/sampling region (two‐way ANOVA: *F*(5, 59) = 4.04, *p* = 0.0032), but not genotype (*F*(2, 59) = 1.07, *p* = 0.35), with no significant interaction between treatment/sampling region and genotype (*F*(10, 59) = 0.37, *p* = 0.95). Post hoc comparisons indicated that SS8 abundance was significantly higher in HT samples than in both recipient boundary and recipient far tissues (*p* = 0.0108 and 0.0085, respectively), while no other pairwise differences among treatment/sampling regions were significant.

**FIGURE 5:**
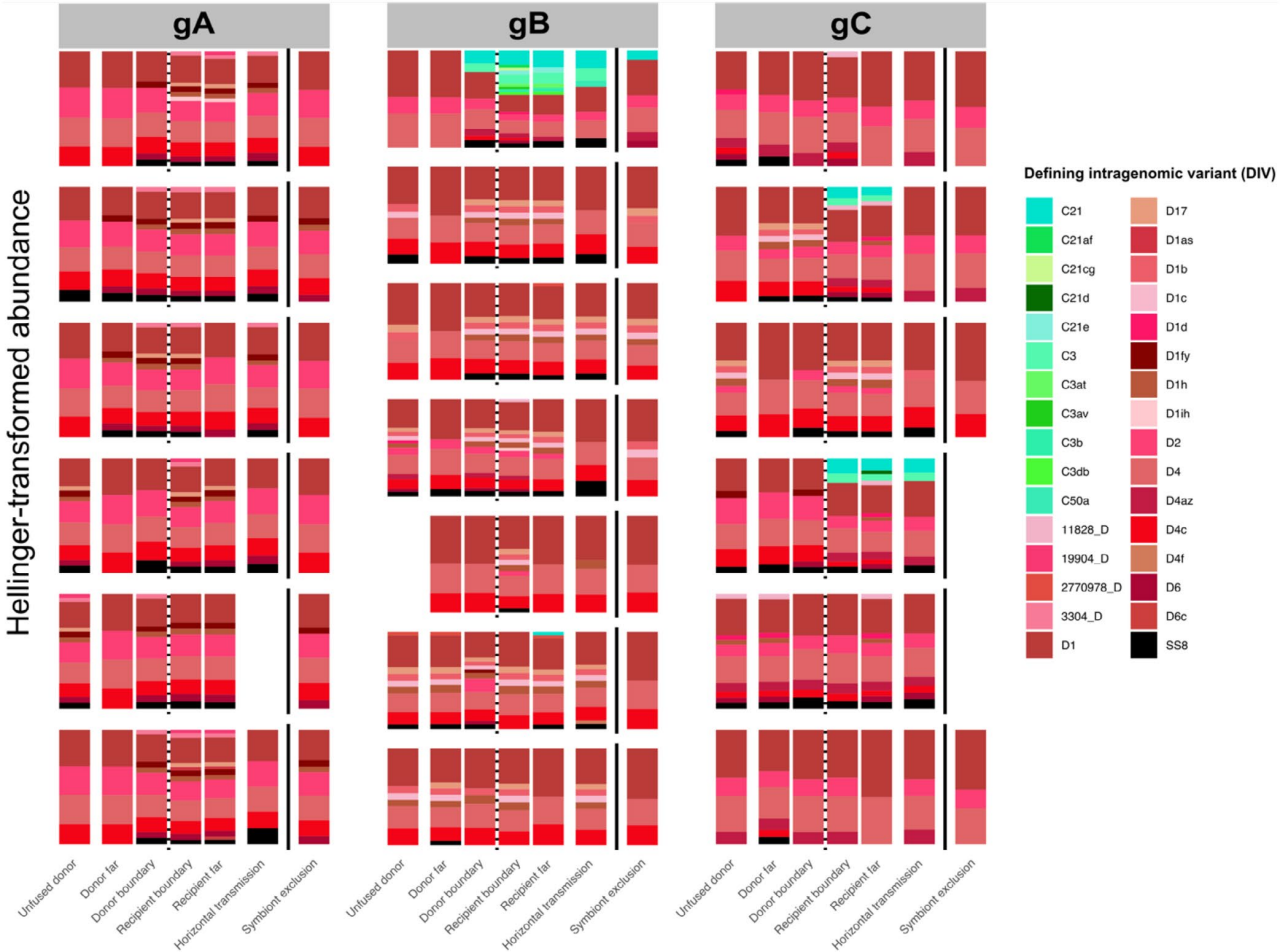
*Galaxea fascicularis* ITS2 Symbiodiniaceae community composition across experimental treatments. Bar plots show ITS2‐derived community profiles at the final timepoint (51 DPF) for three *G. fascicularis* genotypes (gA, gB, and gC). Each plot represents an individual fragment pairing, with bars indicating the proportional abundance of dominant intragenomic variants (DIVs), coloured by genus or identified as SS8 (black). Treatments are faceted as: Unfused donor (UD), donor far, donor boundary, recipient boundary, recipient far, and horizontal transmission (HT) and symbiont‐exclusion (EC) controls. Empty or missing bars indicate samples that did not pass quality control.

## 4 Discussion

This study tested whether adult isograft fusion between healthy ‘donor’ corals hosting heat‐evolved (HE) *Cladocopium proliferum* (strain SS8) and chemically bleached ‘recipients’ could facilitate SS8 acquisition and post‐bleaching recovery. Despite SS8 occurring at low relative abundance (< 1.32%) in *Galaxea fascicularis* donors at the start of the experiment, it was detected in 15 of 19 fused recipients whose paired donor also had detectable SS8 by the final timepoint. However, SS8 was also detected in unfused recipients within the horizontal transmission (HT) control treatment, where its relative abundance was significantly higher than in fused recipients, consistent with previous evidence that corals can acquire expelled symbiont cells from the surrounding water column (Johnston et al. 2025). SS8 detection and relative abundance also showed no consistent spatial gradient between tissues sampled near the fusion interface and those sampled farther from it. This contrasted with the clear spatial pattern in physiological recovery, whereby tissue pigmentation and photochemical efficiency improved more rapidly near the fusion interface in recipients of both *G. fascicularis* and *Psammocora columna*. Together, these findings indicate that adult coral tissue fusion can accelerate spatially localised physiological recovery and coincide with detectable SS8 in recipient tissues. The absence of a corresponding spatial pattern in SS8 abundance, however, indicates that the localised physiological response cannot be attributed solely to the accumulation of SS8 transferred directly across the fusion interface. The results therefore do not fully disentangle direct tissue‐mediated transfer from water‐column acquisition, proliferation of residual recipient symbionts, or other physiological benefits associated with tissue continuity between fused fragments.

### 4.1 Fusion Between Isografts

Of the 52 successful fusion pairings across both species, no instances of incompatible fusion reactions—that is, non‐fusion or tissue necrosis at the interface—were observed throughout the ~50‐day experiment. This outcome was expected, as all fragments in a pair were clonally derived from the same original ‘mother’ fragment and were therefore genetically identical isografts. Indeed, microfragmentation and fusion of clonal fragments is a well‐established aquaculture technique commonly used to accelerate coral growth (Forsman et al. 2006; Page et al. 2018). Although chemical bleaching can impose substantial physiological stress on coral hosts and their symbionts, fusion success was not compromised under the conditions used here.

### 4.2 Fusion to a Healthy Donor Accelerates Bleaching Recovery

Following tissue fusion, the gastrovascular systems (GVS) of the two colonies are expected to integrate (Gateno et al. 1998), potentially facilitating exchange of dissolved and particulate materials, including organic and inorganic nutrients and, in some cases, freely circulating microalgal symbionts (Gateno et al. 1998; Gladfelter 1983). In this study, fused recipients of both species showed visual and physiological patterns in line with localised symbiont recovery near the fusion interface. Macroscopic reflectance imaging revealed greater pigmentation in fused recipients than in unfused controls, with recovery strongest near the fusion boundary and declining with distance from the interface (Figures 2 and 3). Symbiont photochemical efficiency also improved in a similar spatial pattern, suggesting coordinated recovery of both symbiont‐associated pigmentation and photochemical performance (Figure 4A,B). The magnitude of photochemical recovery nevertheless differed between species. Recipient boundary and recipient far tissues increased by 40.9% and 42.6%, respectively, in *P. columna*, compared with 11.6% and 16.2% in *G. fascicularis* . The larger proportional increases in *P. columna* likely partly reflect its lower *F*_*v*_ */F*_*m*_ immediately post‐bleaching, consistent with greater sensitivity to the chemical bleaching treatment. However, interpretation of the mechanisms underlying these patterns is complicated by the nature of chemical bleaching, which removes most—but not all—symbionts from coral host tissues (Scharfenstein et al. 2022). Consequently, it remains unclear whether recovery reflects the enhanced restoration of photochemical function and/or proliferation of residual recipient symbionts, uptake of expelled donor cells, direct transfer through the fused tissue interface, or a combination of these processes. These possibilities are considered below (see Figure 6 for a conceptual overview).

**FIGURE 6:**
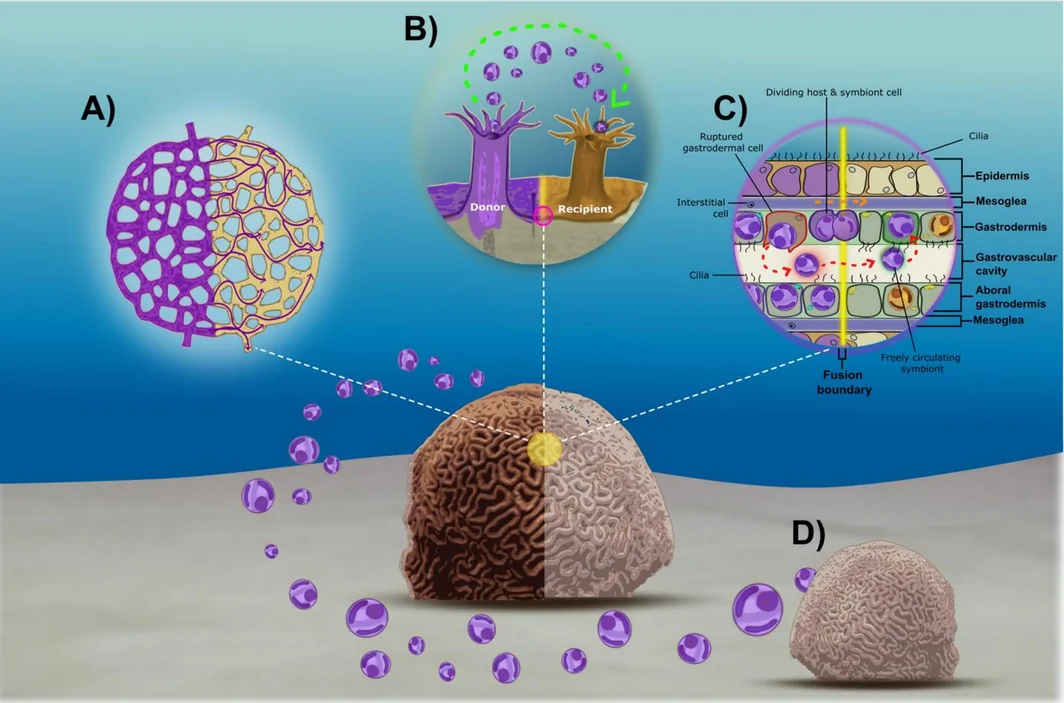
Conceptual diagram illustrating potential mechanisms of heat‐evolved (HE) symbiont and nutrient transfer across coral fragments following tissue fusion. (A) Widespread diffusion of nutrients and metabolites (either passively or via active transport) from donor (purple) to recipient (yellow) across the fusion line; (B) localised expulsion of HE symbionts from donor polyp and subsequent acquisition by recipient at or near the fusion line; (C) integration of the gastrovascular cavities following fusion, potentially allowing donor‐derived symbionts that have been released to circulate through the shared gastrovascular cavity and be acquired by recipient gastrodermal cells (red arrows). Also shown is the potential movement or proliferation of symbiont‐bearing host cells across the fusion line (orange arrows); (D) expulsion of HE symbionts into the water‐column for potential acquisition by neighbouring bleached corals.

### 4.3 Fusion‐Mediated Nutrient Diffusion and Recovery of Residual Symbionts

One possible explanation for the localised recovery observed in fused recipients is that fusion enabled the transfer of donor‐derived metabolites into bleached recipient tissue, supporting host recovery and the proliferation of residual symbionts (Figure 6A). Indeed, previous work in the soft coral *Pseudopterogorgia fulvum fulvum* has shown that, following fusion, ^14^C‐labelled photosynthates can move into the recipient colony and reach tissues up to 390 mm from the fusion line within 24 h (Gateno et al. 1998). Although this study was conducted on a soft coral (Alcyonacea), similar resource‐sharing dynamics may occur in scleractinian corals, including *G. fascicularis* , which possesses a mesh‐like canal system thought to support uniform nutrient transport among polyps (Li et al. 2023). Transferred metabolites could include organic carbon and nitrogenous compounds that support host metabolism and Symbiodiniaceae growth, as ammonium and other host‐derived nutrients are readily assimilated by Symbiodiniaceae and can support protein synthesis and cell division (Rädecker et al. 2021; Yellowlees et al. 2008; Pernice et al. 2012). However, as nutrient fluxes were not measured, metabolite transfer remains a plausible but untested contributor to the observed recovery patterns.

### 4.4 Localised Polyp‐Scale Transfer of Expelled Symbionts From Donor to Recipient

A second, non‐mutually exclusive explanation is that symbionts expelled from donor polyps were locally acquired by adjacent recipient tissue near the fusion interface (Figure 6B). This explanation is consistent with the detection of SS8 in horizontal transmission controls, which indicates that expelled or otherwise released SS8 cells were available in the surrounding water column for acquisition by bleached recipients (Johnston et al. 2025). In fused pairs, the close proximity of donor and recipient polyps may have increased the local density of released symbionts near the fusion boundary, providing a spatially localised source of cells for uptake. Local retention of released symbionts could also be influenced by coral surface flows. The coral surface is surrounded by a diffusive boundary layer (DBL), which can be modified by epidermal ciliary beating to enhance solute transport and retain small particles in the mucus layer (Shapiro et al. 2014; Pacherres et al. 2020; Bouderlique et al. 2022; Selvan et al. 2026). For example, ciliary flow in *Porites lutea* has been shown to entrain suspended particles (~5 μm), concentrating them within the overlying mucus and increasing retention 3.4‐fold (Pacherres et al. 2020). Similar near‐surface flow processes could therefore increase the residence time of expelled symbionts near recipient tissues, particularly where donor and recipient polyps are in direct contact. Interestingly, however, SS8 relative abundance was slightly higher in HT recipients than in fused recipient tissues, indicating no clear enhancement of SS8 abundance associated with proximity to donor polyps. Nonetheless, because expelled‐cell viability, uptake, and functional contribution were not directly measured, this pathway remains a plausible but unresolved contributor to localised recovery.

### 4.5 Fused Donor to Recipient Symbiont Migration

A third, non‐mutually exclusive explanation for the earlier and more localised recovery observed in fused recipients is that tissue fusion may have permitted symbionts to move directly through newly connected gastrovascular or gastrodermal tissues (Figure 6C; red arrow). Freely circulating Symbiodiniaceae cells have been directly observed within the coelenteron of reef‐building corals, including *Acropora cervicornis* (Gladfelter 1983), as well as in several octocoral species, where such movement has been proposed to contribute to bleaching recovery (Parrin et al. 2016). Under this scenario, donor‐derived symbionts released into a shared gastrovascular cavity could move into recipient regions where they may be captured by ciliated endodermal cells—a process consistent with previously described mechanisms of symbiont uptake in corals (Schwarz et al. 1999).

In addition to movement via the gastrovascular cavity, localised recovery may reflect redistribution of symbionts through newly connected gastrodermal tissue, potentially involving movement or proliferation of symbiont‐bearing host cells across the fusion interface (Figure 6C; orange arrow). This possibility is supported by observations from Bockel and Rinkevich (Bockel and Rinkevich 2019), who documented rapid migration of endosymbiotic dinoflagellates from densely populated tissues into newly developed, symbiont‐free tissue in Red Sea pocilloporid corals. However, the precise cellular mechanisms underlying rapid symbiont movement through host tissues remain largely unknown.

It is important to note that the above mechanisms are not mutually exclusive and may operate sequentially or synergistically. Donor‐derived metabolites could enhance host condition and support the proliferation of residual symbionts, ciliary modification of the DBL could increase local retention and uptake of expelled cells near the fusion interface, and gastrovascular integration could provide an additional internal route for symbiont transport following tissue fusion. However, because nutrient fluxes, near‐surface hydrodynamics, and symbiont movement were not measured directly, the relative and interactive contributions of these mechanisms cannot be distinguished from the present data.

Although this study primarily examined the impact of fusion on the bleached recipient, fusion could also be expected to affect the donor if nutrients or symbionts are shared with an attached, resource‐limited partner. However, both reflectance and photochemical efficiency were indistinguishable between fused and unfused donor corals, suggesting no detectable physiological cost to the donor under the experimental conditions and timeframe here. This apparent asymmetry may reflect a broader principle of modularity in colonial organisms, in which the ability to integrate additional units at minimal physiological cost represents a key evolutionary advantage of the coral colonial lifestyle.

### 4.6 Future Directions

Several important questions remain before the broader ecological relevance of fusion‐mediated recovery and symbiont acquisition can be established. First, future work should determine whether similar patterns occur in allografts between genetically distinct conspecifics, or in xenografts between closely related species. Determining this will be critical for assessing the ecological relevance of this method. Although allograft and xenograft pairings often lead to tissue rejection or necrosis, Nozawa and Loya (Nozawa and Loya 2005) showed that juvenile *Seriatopora* allografts and xenografts can exhibit signs of early‐stage fusion that only later transition into tissue separation or necrotic disconnection as the allorecognition system matures. Whether a comparable window of transient fusion exists in adult corals with more developed allorecognition systems remains unknown; however, even short periods of tissue integration may be sufficient to permit resource exchange or symbiont movement across the fusion boundary.

Future work should also determine whether low‐abundance SS8 contributes measurably to coral performance. In the present study, SS8 was detected in fused *G. fascicularis* recipients, but its relative abundance was generally low and its functional contribution to photochemical recovery could not be separated from residual recipient symbiont proliferation or donor‐derived nutritional support. Controlled dose–response experiments, longer‐term monitoring, and subsequent heat‐stress assays will be needed to test whether acquired HE symbionts can meaningfully alter post‐bleaching recovery or thermal tolerance.

A major challenge in studies of symbiont transmission will be disentangling the mechanisms by which introduced symbionts arrive and persist within recipient tissues. Specifically, future studies will need to control for potential horizontal acquisition from the surrounding water column in order to distinguish this pathway from direct symbiont transfer across the fusion interface. One potential approach would be to develop an agarose‐based system designed to restrict the dispersal of expelled symbionts and reduce their transport by coral‐generated ciliary currents. By limiting water movement around the fragments while preserving direct tissue contact at the fusion interface, such a system could provide a more stringent test of whether symbionts move directly between fused partners. Residual symbiont proliferation represents another possible explanation for recipient recovery and will also require further examination. Live‐cell imaging at the fusion line, particularly where donor‐ and recipient‐derived symbionts can be differentiated, could help resolve the real‐time dynamics of symbiont movement, cellular division, and incorporation into recipient tissues. In addition to symbiont acquisition and proliferation, nutrient exchange between fused partners may also contribute to recovery. Stable isotope labelling of donor colonies, such as with ^13^C‐bicarbonate or ^15^N‐nitrate, could be used to assess the direction, extent, and spatial distribution of nutrient flow across the fusion interface. Although this approach would not resolve symbiont identity, it would help clarify whether localised metabolic enrichment contributes to recipient recovery.

A further limitation of the present study is the lack of tank replication across SS8‐exposed and symbiont‐exclusion controls. Because each group was housed in a single tank, SS8‐related effects cannot be fully separated from other tank‐specific differences in environmental conditions or husbandry. Replication of both groups across multiple independent tanks would therefore be required to attribute observed differences more confidently to SS8 exposure rather than tank‐level variation.

## 5 Conclusion

This study provides a controlled first test of adult isograft fusion as a potential pathway for localised recovery and acquisition of experimentally evolved heat‐tolerant Symbiodiniaceae. Fusion with SS8‐associated donor fragments accelerated pigmentation recovery and enhanced photochemical efficiency in chemically bleached recipients, with recovery strongest near the fusion interface. In *G. fascicularis* , SS8 was detected in most fused recipients, consistent with HE symbiont acquisition, although its presence in horizontal transmission controls indicates that water‐column uptake also contributed. These findings suggest that adult coral tissue fusion can support localised post‐bleaching recovery and may contribute to symbiont movement between coral fragments. However, the relative contributions of direct tissue‐mediated transfer, water‐column acquisition, residual symbiont proliferation, and donor‐derived nutrient exchange remain unresolved.

## Author Contributions

**Bede G. Johnston:** conceptualization, investigation, writing – original draft, methodology, validation, visualization, writing – review and editing, formal analysis, project administration, data curation. **Catalina Parra V.:** conceptualization, investigation, methodology, project administration. **Matthew R. Nitschke:** conceptualization, investigation, funding acquisition, methodology, writing – review and editing, supervision, resources, project administration. **Wing Yan Chan:** supervision, conceptualization, investigation. **Madeleine J. H. van Oppen:** conceptualization, investigation, funding acquisition, methodology, project administration, supervision, formal analysis, resources.

## Funding

This work was supported by the Paul G. Allen Family Foundation, Australian Research Council (FL180100036), Reef Restoration and Adapation Program (RRAP) is a partnership between the Australian Governments Reef Trust and the Great Barrier Reef Foundation, Australian Research Council Discovery Early Career Researcher Award (DE240100317), Royal Society Te Apārangi Marsden Fast Start (21‐VUW‐021), Westpac Research Fellowship, Allen Family Philanthropies, University of Melbourne.

## Conflicts of Interest

The authors declare no conflicts of interest.

## Supporting information


**Figure S1:** Experimental setup of *Galaxea fascicularis* in a 100 L tank containing both fused and unfused donor–recipient pairs. Corals assigned to the symbiont exclusion (EC) treatment were housed separately and are not shown in this tank.
**Table S1:** Days required for succesful fusion and tissue integration to be observed for each donor‐recipient fusion pairing. Fusion was considered to have occured when the tissues of adjoining fragments had visibly merged in at least once location at the boundary and tissue origin was no longer distinguishable.
**Figure S2:** Representative image of a fused *Psammocora columna* pair, showing the symbiont‐rich donor fragment on the left (blue‐green) and the bleached recipient fragment on the right. The image depicts spatial variation in photochemical efficiency across the fusion boundary, as visualised using PAM fluorescence imaging. Red scale bar = 1 cm.
**Figure S3:** Workflow for tissue extraction from fused donor–recipient coral pairs. (A) Fused pair is first removed from the plug. (B) Donor and recipient fragments are then separated along the fusion line. (C) The recipient fragment is subdivided into two regions: boundary (adjacent to the fusion line, following a ~1 mm tissue shave) and far (distal from the fusion site). Donor subdivision not shown. (D) Generation of triplicate tissue samples from each region: recipient far (left) and recipient boundary (right).
**Table S2:** Instances of SS8 DIV occurrences by category combination in *Galaxea fascicularis* .
**Figure S4:** *Galaxea fascicularis* ITS2 Symbiodiniaceae community composition in experimental donor fragments prior to the start of the experiment (0 DPF). Bar plots show Hellinger‐transformed ITS2‐derived symbiont communities for three *G. fascicularis* genotypes (gA, gB, and gC). Each panel represents an individual fragment, with bars indicating the proportional abundance of dominant intragenomic variants (DIVs), coloured by genus or identified as SS8 (black).
**Figure S5:** *Galaxea fascicularis* ITS2 Symbiodiniaceae community composition in experimental recipient fragments pre and post chemical bleaching. Bar plots show Hellinger‐transformed ITS2‐derived symbiont communities for three *G. fascicularis* genotypes (gA, gB, and gC). Each panel represents an individual fragment, with bars indicating the proportional abundance of dominant intragenomic variants (DIVs), coloured by genus.

## Data Availability

Raw ITS2 Symbiodiniaceae sequences are available in GenBank (BioSample accession nos.: SAMN63302893 − SAMN63303441; BioProject ID: PRJNA1533200).
